# Obesogenic diet and metabolic syndrome among adolescents in India: data-driven cluster analysis

**DOI:** 10.1186/s12872-023-03429-y

**Published:** 2023-08-09

**Authors:** Kirti Kirti, Shri Kant Singh

**Affiliations:** Department of Survey Research and Data Analytics, International Institute for Population Sciences, Govandi Station Road, Deonar, Mumbai, Maharashtra 400088 India

**Keywords:** Obesogenic diet, K-means algorithm, Adolescents, Metabolic syndrome, Micronutrient deficiencies

## Abstract

**Background:**

Metabolic syndrome is on the rise in India and is primarily linked to obesogenic dietary habits. The synergy of both is a prominent risk factor for cardiovascular diseases (CVDs). Hence, the present study aims to unveil clusters at high risk of metabolic syndrome and ascertain cluster characteristics based on dietary patterns among adolescents aged 10–19 years.

**Data and methods:**

The study utilizes secondary data, i.e., Comprehensive National Nutrition Survey conducted in 2016-18. The study sample includes children and adolescents aged 10–19 years. An unsupervised learning algorithm was used to ascertain possible clusters in the data based on individuals’ dietary patterns. The k-means were used to cluster the data according to their dietary patterns. To determine the number of clusters elbow method was used, and appropriate validation indices were also obtained for the final k. Further, to ascertain the distribution of the obesogenic dietary patterns and metabolic conditions in each cluster was analysed. Bivariate descriptive analysis was used to draw further inferences.

**Results:**

The k-means clusters identified five optimum clusters based on 12,318 adolescents (6333 males (mean age:14.2 ± 2.8) and 5985 females (mean age:14.3 ± 2.8)) 17 dietary patterns. Clusters were named based on how prudent these were in terms of consuming a healthy diet. Cluster phenotypic characteristics were defined as follows: a cluster of obesogenic diets (24%) constituted the highest proportion of the total sample and was significantly suffering from obesity (p < 0.001), and greater proportions of lipid anomalies (p = 0.51) and hypertension (p = 0.44) but not statistically significant. In contrast, 21% of the sample comprised a plant-based diet cluster and suffered from all deficiencies but folate (p = 0.625), zinc (p = 0.132), and greater proportion from obesity (p = 0.19; not significant), and diabetes (p < 0.001). A cluster of “convenient” (20%) mainly suffered from lipid anomalies (p = 0.00), diabetes (p = 0.03), and a greater proportion from hypertension (p = 0.56) with deficiencies of all the essential vitamins and minerals but significantly from vitamin A (p < 0.001), folate (p < 0.001), and iron (p = 0.017). Lastly, the cluster of those who follow a “Western diet” (17%) was found to have lipid anomalies (p = 0.003), diabetes (p = 0.016), greater proportion of vitamin B12 (p = 0.136), D (p = 0.002), folate (p < 0.001), and iron deficiencies (p = 0.013).

**Conclusions and relevance:**

Adolescents in India show a strong association between obesogenic diet and metabolic syndrome. Therefore, the burden of metabolic syndrome at early ages can be prevented by controlling obesogenic dietary practices and addressing micronutrient deficiencies. This may be done by targeted health promotional campaigns in schools and college-going populations in India.

**Table:** 

Contributions to the literature	
1.	The present study unveils potential clustering among adolescents aged 10-19 years in India based on their dietary patterns is the first in India among adolescents.
2.	Individuals following a highly prudent obesogenic diet were found to have a significant association with two major components of metabolic syndrome i.e., obesity and diabetes.
3.	Lastly, individuals who consume less cereal, and milk paired with occasional consumption of fats, sugar, and the rest of the food groups, and follow a mainly non-vegetarian diet, were found to have iron and zinc deficiencies. Additionally, they had all components of metabolic syndrome more prominent than the rest of the clusters.

## Background

Metabolic syndrome (MetS) is an amalgamation of significant cardiovascular disease (CVD) risk factors: obesity, high cholesterol, diabetes and pre-diabetes, and high blood pressure [1, 2]. In India, the prevalence of MetS ranges from 4 to 41% among various subgroups of populations and regions [3]. Hence, emerging as a leading cause of premature deaths globally [4–6]. However, evidence suggests that the co-occurrence of high-risk behavioral factors, such as lack of physical activity, unhealthy diet, tobacco and alcohol consumption, yields greater risks of chronic diseases and metabolic syndrome [4, 7, 8]. However, dietary intake alone contributes the highest to the prevalence of diet-related non-communicable diseases (NCDs) and other chronic conditions [8–11]. Such dietary habits in early life might disrupt the overall well-being of an individual and hasten the progression of chronic illnesses later in life [10, 12–14]. Considering the current state of health transition attributable to the epidemiological shift in India, i.e., the increased burden of metabolic syndrome owing to lifestyle changes, changes in dietary patterns, and increasing urbanization is projected to be doubled in a decade [11, 12].

It is profound that food impacts the imbalance or absence of vital nutrients and minerals that significantly affect everyday performance, behavior, mood, and cognitive, and physical activities [15]. Whilst, individuals in their early ages are more prone to develop various unhealthy lifestyles including inappropriate dietary habits and other behavioral aspects that are pushing them towards developing serious chronic conditions at later ages [16]. Hence, increased intake of energy-dense foods that are high in fat and sugars paired with declined physical activity due to a sedentary lifestyle and increasing urbanization lead to metabolic syndrome in all ages, particularly affecting adolescents’ health [15]. This high caloric intake coexisting with a nutrient deficiency in an individual would lead to inefficient utilization of calories. Toxic by-products of incomplete biochemical processes produce a vicious loop that leads to more weight gain, depression, eating disorders, metabolic syndrome, exhaustion, and other typical symptoms. However, there is a great dearth in evidence towards explaining how consuming a particular food group can lead to metabolic syndrome in an individual.

Further, high caloric intake increase in the prevalence of obesity and lipid anomalies in early ages has led to increased prevalence of diabetes, hypertension at later ages in India over time and can be linked with multimorbidity in an individual [17]. Which, certainly, cannot be neglected. There is a wide gap in the prevailing knowledge pertaining to how obesogenic diet would be related to the risk of metabolic syndrome among adolescents in India as a whole. There is no such study in India where this pertinent issue has been addressed in a way that it will aid current clinical practices at low cost in the treatment of MetS among younger generation. Further, the present study emphasises on how pertinent it is to give utmost attention to the increasing burden of early-onset of MetS attributable to diet and prevent adverse effect of such conditions in the later ages. Adverse effect could range from individual health to economic burden due to multimorbidity, and perhaps tempering with the quality of life too [18–20].

Therefore, methodologically, the present study utilises unsupervised learning which helped in addressing the multi-dimensionality of the dietary patterns hence, its relation with metabolic syndrome and micronutrient deficiencies. Exploring dietary behaviour among adolescents at a national level and how it is linked with metabolic syndrome and micronutrient deficiencies has not been done in any of the studies done previously in India. As, prolonged consumption of certain food groups might accelerate the onset of adverse health outcome. Therefore, the present study tried to unveil potential clustering among adolescents aged 10–19 years in India based on their dietary patterns. Further, exploring how each component of metabolic syndrome and deficiencies of essential vitamins and minerals was distributed across each cluster. Finally establishing association between metabolic syndrome and various micronutrient deficiencies in each cluster along with the strength of its association.

## Methods

### Setting, sampling technique, and participants

The present study utilises secondary data from the “Comprehensive National Nutrition Survey (CNNS) (2016-18)” under the supervision of the Ministry of Health and Family Welfare, Government of India, in collaboration with UNICEF, conducted by the Population Council. CNNS is India’s first-ever nationally representative nutrition survey of children and adolescents. The CNNS selected a representative sample of households and individuals across the 30 states using a multi-stage sampling design. The rural sample was chosen in two stages in each state. The first stage involved selecting PSUs via probability proportional to size (PPS) sampling, followed by a systematic random selection of households within each PSU. The sampling design in large PSUs consisted of three stages, with the addition of a segmentation procedure to reduce enumeration areas to manageable sizes. However, in urban areas, the sampling frame includes two stage sampling, first the PPS in urban wards followed by random selection of census enumeration blocks (CEB) from each ward [21].

In addition, the survey collected data on anthropometric and NCDs among individuals aged 5–19 years. However, the present study utilises information on those in the ages 10–19 years. The data can be obtained by reasonable request to the population Council website: https://www.popcouncil.org/uploads/pdfs/2019RH_CNNSreport.pdf.

The CNNS used a multi-stage sampling design to select a representative sample of households and individuals aged 0–19 years across the 30 states. In each state, the rural sample was selected in two stages. The first stage was the selection of PSUs using probability proportional to size (PPS) sampling and the second stage was a systematic random selection of households within each PSU. In large PSUs, the sampling design involved three stages, with the addition of a segmentation procedure to reduce enumeration areas to manageable sizes.

### Final study population after data cleaning and preparation

**Fig. 1:**
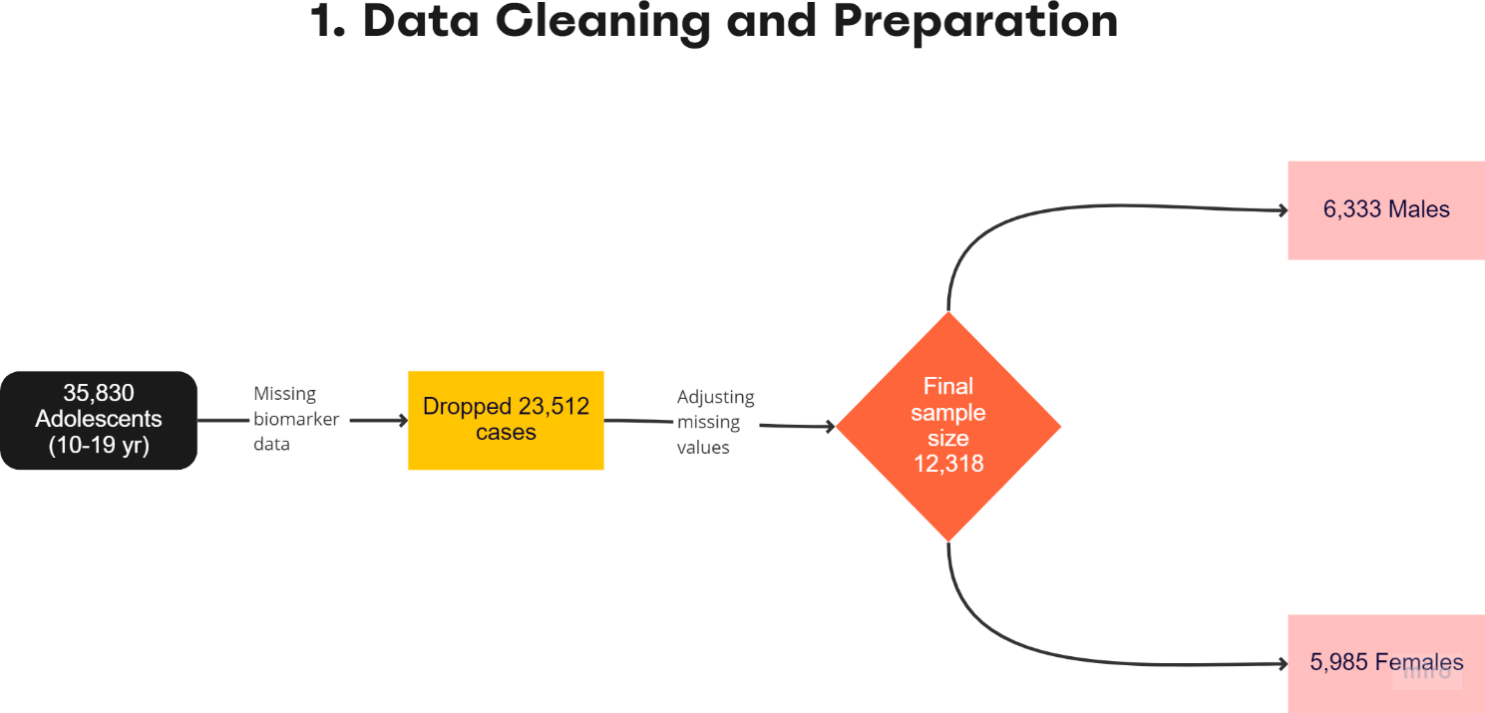
Flowchart showing derivation of study sample

As per WHO definition, the age range for adolescents is between 10 and 19 years [22]. Therefore, the study population for the present research utilised information among children and adolescents aged 10–19 years. Hence, a sample of 12,318 adolescents aged 10–19 years where biomarkers were recorded were taken into consideration. With 6,333 male and 5,985 female adolescents as shown in Fig. 1.

### Derivation of measures used for evaluation of clusters

#### Obesogenic diet

In CNNS, seventeen questions pertaining to dietary practices were asked, how frequently they consuming a particular food group in a week which includes cereals/milk/pulses/greens/roots/vegetables/fruits/eggs/fish/chicken/nuts and oilseeds/fats and oils/sugar jaggary/fried foods/junk foods/sweets/aerated drinks in a week. Those who consume fried/aerated/junk food more frequently considered as obesogenic diet. In contrast, those who consume less frequently, say cereal, were considered as obesogenic diet. For instance, “7” corresponds to those who “daily consume, say, cereal,” which can be considered as a good eating habit. Whereas, in the case of junk/sweet/fried etc. food, “0” were those who “never consume junk food,” which is again a good thing. Similar re-coding was done for the remaining fifteen items, k-means clustering algorithm was then used after checking the clustering tendency or randomness of the data.

##### Metabolic syndrome

Four components of metabolic syndrome include obesity, lipid abnormality, diabetes, and hypertension.

#### Overweight/Obesity

Body mass index for age z-score was used for categorizing individual as “non-overweight/obese” (BMI ≤ + 1SD) and “overweight/obese” (BMI > + 1 SD) group. The categorisation was based on Asian criterion, as it is different than other continents [9, 23].

#### Type 2 diabetes

Adolescents with pre-diabetes/diabetes were determined using glycosylated hemoglobin (HbA1c) [16]. According to the American Diabetes Association recommendation, glycated hemoglobin (HbA1c) can be used as a proxy to fasting blood glucose for the diagnosis of type 2 diabetes [24]. Cut-off points was considered as pre-diabetes (lying between 5.6% and 6.4%) and diabetes (greater than 6.4%) [10, 21, 25]. The final variable was then categorized into 0 “no pre-diabetes or diabetes” and 1 “pre-diabetes/diabetes.”

#### Hypertension

Individuals with systolic blood pressure greater than 139 mmHg or diastolic blood pressure greater than 89 mmHg were coded as “1” i.e., hypertensives and “0” as non-hypertensives.

#### Lipid anomaly

Lipid profile primarily includes total serum cholesterol (assessed by spectrophotometry using cholesterol oxidase esterase peroxidase in CNNS), high-density lipoprotein cholesterol (HDL-C) (assessed by spectrophotometry and direct measure polyethylene glycol-modified cholesterol oxidase), low-density lipoprotein cholesterol (LDL-C) (assessed by spectrophotometry and direct measure cholesterol oxidase), and triglycerides (assessed by spectrophotometry and enzymatic end point method).

Serum triglycerides was categorized into “low” (< 130 mg/dl) and “high/borderline” (≥ 130 mg/dl), cholesterol was recoded as “low” (< 200 mg/dl) and “high/borderline” (≥ 200 mg/dl), low-density lipoprotein (LDL) was categorized as “low” (< 130 mg/dl) and “high/borderline” (≥ 130 mg/dl), and high-density lipoprotein (HDL) cholesterol was recoded as “borderline/low” (< 40 mg/dl) and “high” (≥ 40 mg/dl).

Further, whether an individual was suffering from any lipid anomalies, or no lipid anomalies, were derived from the combination of whether an individual was suffering from high total cholesterol, high LDL-C, high triglycerides, or low HDL-C. Therefore, they were categorized into two groups “0”: no lipid anomalies and “1”: any lipid anomalies.

#### Micronutrient levels

Vitamin A deficiency was categorized as “yes” (serum retinol concentration < 20 µg/dL) else “no”. Vitamin B12 deficiency was categorized into “yes” (serum vitamin B12 < 203 pg/ml) else “no”. Vitamin D deficiency was categorized into “yes” (serum 25 (OH) concentration < 12ng/mL (30 nmol/L)) else “no”. Folate deficiency was categorized into “yes” (serum erythrocyte folate < 151ng/ml) else “no”. Iron deficiency was categorized into “yes” (serum ferittin < 15 µg/l) else “no”. Zinc deficiency was categorized into “yes” (Serum zinc concentration < 70 µg/dl (morning fasting) and < 66 µg/dl (morning non-fasting) in non-pregnant females and < 74 µg/dl in males). Iodine status was categorized into “Adequate” (median urinary iodine concentrations (mUIC) > 100 µg/L and < 300 µg/L) and “Suboptimal” (mUIC < 50 µg/L).

#### Other variables

The age considered was considered as continuous variable aged 10–19 years. Gender as “male” and “female.”

### Analytical process

#### Prerequisites

##### i. Cluster tendency

Firstly, the clustering tendency or randomness of a data set was measured before subjecting the data to a clustering algorithm using Hopkins statistics denoted by ‘H’ [26]. The statistics was based on the difference between the distance from a real point to its nearest neighbour, U, and the distance from a randomly chosen point within the data space to the nearest real data point, W. Value for ‘H’ higher than 0.70 indicates that the data has a high tendency to form clusters [26].

##### ii. Obtaining the optimum-value of k

Using the combination of the elbow method, distortion plot, and silhouette score optimum value of k was obtained [27]. Only after obtaining an optimum value of k, k-means algorithm was used.

#### K-means clustering algorithm

Prior to applying the k-means algorithm to cluster the adolescents, it was also made sure that the information was complete and there was no missing data. Further, after analysing the clustering tendency of the data and obtaining the optimum value of k, k-means algorithm was used to finally cluster the data into k clusters using the ‘sklearn’ library in Python [28]. Clustering was done based on seventeen available dietary items available in order to diminish subjectivity of these items. Evaluation of each cluster by background characteristics, using descriptive analyses was done. Phenotypic characteristics of each cluster was also analysed. Further, distribution of each component of metabolic syndrome and micronutrient deficiencies within all five clusters was analysed. Lastly, association of any metabolic syndrome and micronutrient deficiencies with clusters were also explored using chi square test for association.

The study followed the STrengthening the Reporting of Observational studies in Epidemiology (STROBE) reporting guidelines for cross-sectional study (Additional file 1). National weights of biomarker have been used while exploring and computational analysis. STATA(SE) version 16.0 software has been used for data analysis and data wrangling and “sklearn” library in python was used to perform k-means cluster analysis.

## Results

The results obtained were based on a nationally representative CNNS dataset conducted in the year 2016-18. Due to its sampling design which was multistage, stratified, PPS cluster sampling to recruit sample units, hence the sample estimates were reliable and can be generalised at national level. From Table 1, background characteristics of the study sample and how the prevalence of metabolic syndrome is distributed and associated with gender can be obtained. Approximately, 51% of the study were male (mean age = 14.2; SD = 2.8) and 49% were female adolescents (Mean age = 14.4; SD = 2.8). The mean age can be seen significantly different among male and female (at 5% significance level). Pre-diabetes/diabetes (p = 0.006) and lipid anomalies (p < 0.001) can be seen significantly associated with the gender of the individual. However, the prevalence of overweight/obesity (chi-squared p = 0.105) and pre-diabetes/diabetes (p = 0.006) among males was slightly higher with a prevalence of approximately 13.13% (SD = 0.45) and 12.55% (SD = 0.44) respectively. Whereas among female sample it was 11.63% (SD = 0.43) and 9.43% (0.39) respectively with a total prevalence of 12.40% (SD = 0.32) and 11.02% (SD = 0.30) respectively. Similarly, the prevalence of any lipid anomalies and hypertension was higher among female with the prevalence of 71.13 (SD = 0.61) and 3.64 (SD = 0.25) respectively. However, among men the same prevalence was found to be 67.53 (SD = 0.62) and 2.17% (SD = 0.18) respectively. However, hypertension (p = 0.542) was not significantly associated with the gender of the individual.

**Table 1:** Sample characteristics along with t-test and chi-square test (wherever applicable) for group (gender) difference/association

	Male	Female	Total	p-value (t-test/chi^2^ test)
**N (%)**	6333 (51.41)	5985 (48.59)	12,318 (100)	N/A
**Mean age (SD)**	14.20 ± 2.80	14.30 ± 2.80	14.25 ± 2.80	0.048
**Overweight/Obesity** **(% (SD))**	13.13 ± 0.45	11.63 ± 0.43	12.40 ± 0.32	0.105
**Pre-diabetes/diabetes (% (SD))**	12.55 ± 0.44	9.43 ± 0.39	11.02 ± 0.30	0.006
**Lipid anomaly (% (SD))**	67.53 ± 0.62	71.13 ± 0.61	69.29 ± 0.44	< 0.001
**Hypertension (% (SD))**	2.17 ± 0.18	3.64 ± 0.25	2.89 ± 0.16	0.542

**Fig. 2:**
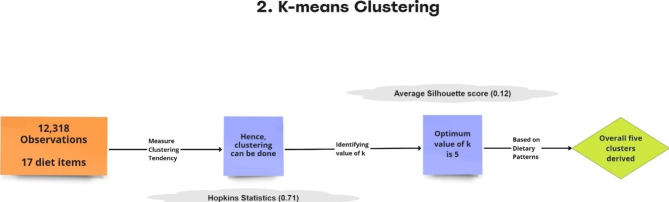
K-means algorithm workflow

The k-means cluster analysis was done based on individuals’ dietary behaviour, with a k value of 5 using k-means algorithm (max iteration = 1000) in Python 3 on CNNS survey dataset. The workflow can be seen in Fig. 2. Five disjoint clusters were discovered after performing the k means cluster analysis on scaled and centred values of 17 dietary items.

**Fig. 3.1:**
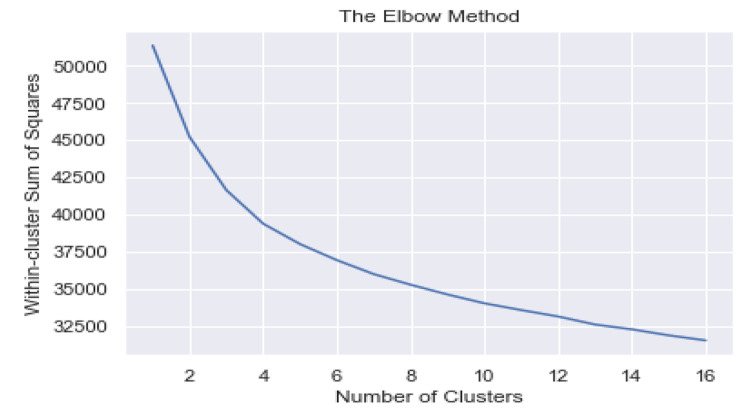
Elbow plot showing within cluster sum of squares (WCSS) over values of k

**Fig. 3.2:**
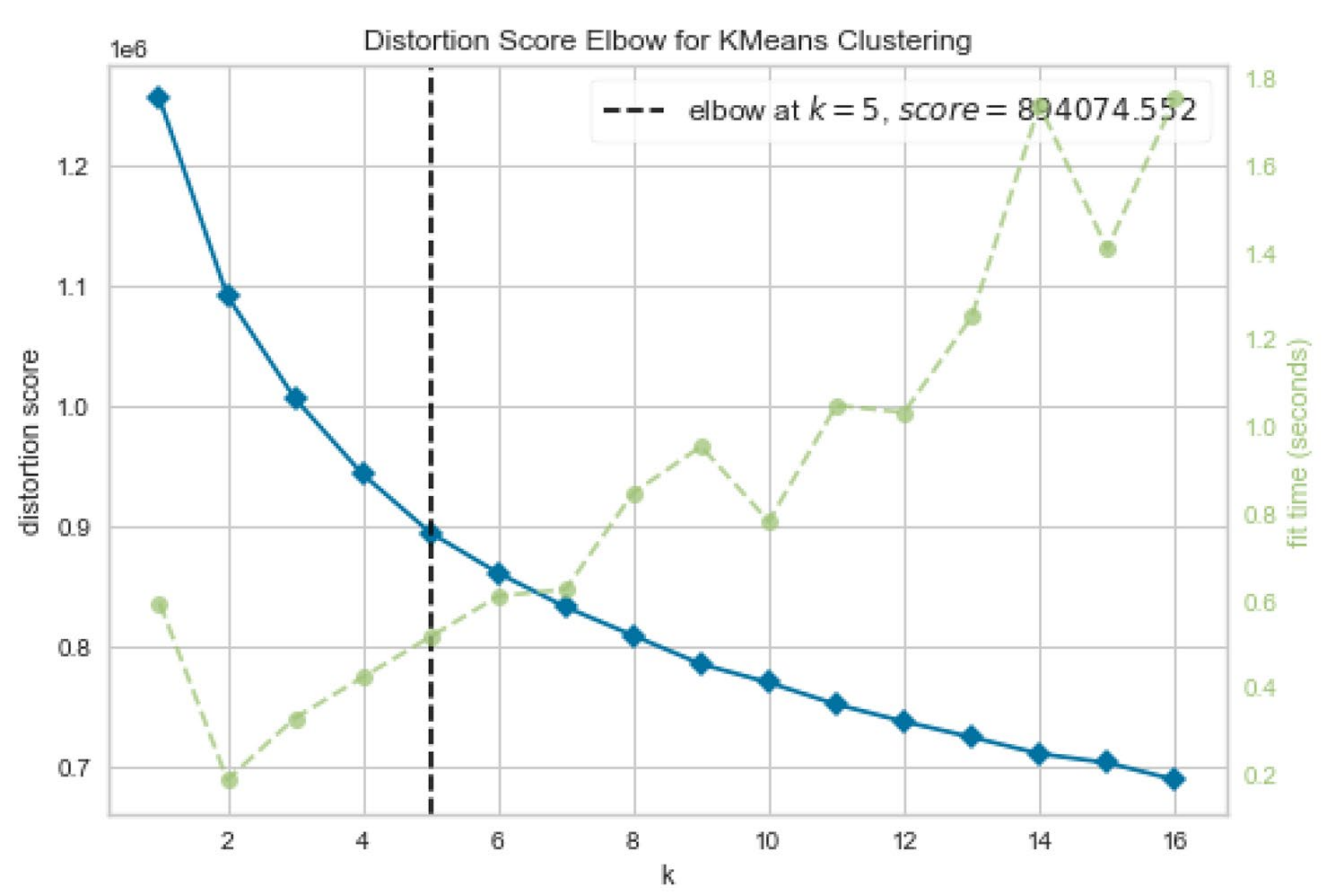
Distortion plot showing distortion score over values of k

Cluster forming tendency of the data was validated by the Hopkins statistic value (H = 0.71). The optimal number of clusters was determined based on silhouette-score (with average Silhouette score = 0.12) and a using a distortion plot. Figure 3.1 depicts a line graph known as an elbow plot of within cluster sum of squares over various values of k along with the distortion plot (Fig. 3.2) showing the optimum value of k. Indicating that the degree of decline in the variation for consecutive values of k decreases and saturates approximately when k is equal to five, i.e., it can be inferred that there exist approximately five heterogeneous clusters among adolescents in the ages 10–19 years based on their dietary pattern.

**Fig. 4:**
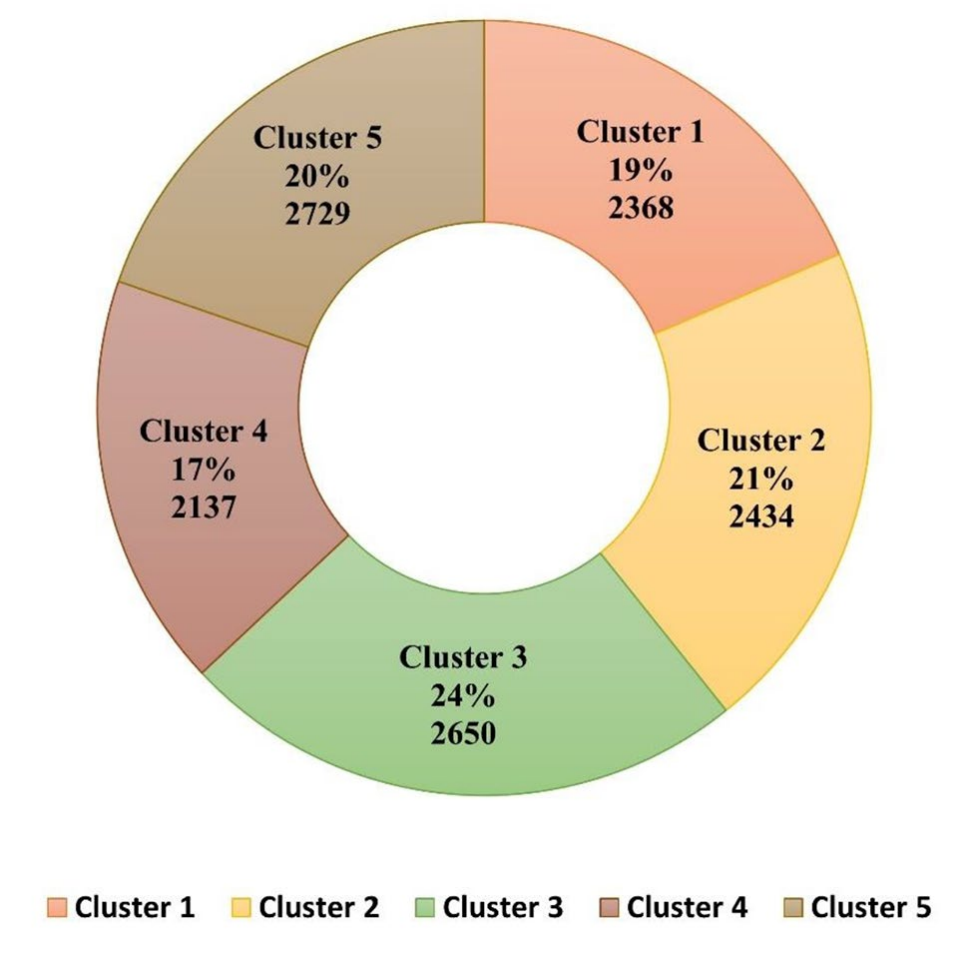
Cluster sample distribution

Figure 4 shows the cluster-wise sample distribution. Distribution of 17 dietary items in accordance with its consumption among individuals in a week across five identified clusters can be seen in Table 2, gives insight into phenotypic characteristics of each cluster. Further, evaluation includes distribution of metabolic syndrome and micronutrient deficiencies can be found in Table 3.1, 3.2, 4.1, and 4.2 provided along with their association with predicted clusters in respective tables.

**Table 2:** Distribution of 17 dietary items according to individuals’ frequencies of consumption in a week across five identified clusters among adolescents aged 10–19 years in India (2016-18)

Cereals	Cluster 1	Cluster 2	Cluster 3	Cluster 4	Cluster 5
Frequently	18.36 ± 0.39	20.60 ± 0.40	22.38 ± 0.42	17.67 ± 0.38	20.99 ± 0.41
Occasionally	19.66 ± 1.19	21.89 ± 1.24	35.14 ± 1.43	14.38 ± 1.05	8.94 ± 0.86
Never	19.76 ± 4.84	24.66 ± 5.24	40.89 ± 5.98	9.33 ± 3.54	5.36 ± 2.74
**Milk**					
Frequently	3.71 ± 0.25	38.54 ± 0.64	24.97 ± 0.57	32.65 ± 0.62	0.12 ± 0.05
Occasionally	34.26 ± 0.70	2.04 ± 0.21	22.94 ± 0.62	1.02 ± 0.15	39.74 ± 0.73
Never	34.59 ± 1.64	0.00 ± 0.00	19.96 ± 1.38	0.00 ± 0.00	45.46 ± 1.72
**Pulses**					
Frequently	13.70 ± 0.51	18.07 ± 0.57	24.73 ± 0.64	19.64 ± 0.59	23.86 ± 0.63
Occasionally	21.93 ± 0.52	22.72 ± 0.52	22.68 ± 0.52	15.85 ± 0.45	16.81 ± 0.47
Never	16.90 ± 3.20	17.15 ± 3.22	42.35 ± 4.22	6.90 ± 2.16	16.72 ± 3.18
**Greens**					
Frequently	16.30 ± 0.60	20.42 ± 0.65	23.10 ± 0.68	20.23 ± 0.65	19.95 ± 0.65
Occasionally	19.82 ± 0.47	20.96 ± 0.48	23.75 ± 0.50	15.70 ± 0.43	19.78 ± 0.47
Never	11.76 ± 2.50	19.39 ± 3.06	39.64 ± 3.79	18.78 ± 3.03	10.44 ± 2.37
**Roots**					
Frequently	17.71 ± 0.55	17.20 ± 0.54	22.62 ± 0.60	17.70 ± 0.55	24.78 ± 0.62
Occasionally	19.21 ± 0.51	23.59 ± 0.54	24.11 ± 0.55	17.14 ± 0.48	15.94 ± 0.47
Never	16.22 ± 2.28	20.04 ± 2.47	36.71 ± 2.98	13.32 ± 2.10	13.71 ± 2.12
**Vegetables**					
Frequently	13.28 ± 0.56	20.42 ± 0.67	24.56 ± 0.71	17.70 ± 0.63	24.04 ± 0.71
Occasionally	21.24 ± 0.48	21.06 ± 0.48	22.93 ± 0.49	17.02 ± 0.44	17.75 ± 0.45
Never	13.94 ± 2.48	15.26 ± 2.58	39.86 ± 3.51	19.87 ± 2.86	11.08 ± 2.25
**Fruits**					
Frequently	6.04 ± 0.59	29.56 ± 1.13	25.64 ± 1.08	25.93 ± 1.08	12.83 ± 0.83
Occasionally	20.53 ± 0.42	19.42 ± 0.41	23.13 ± 0.44	16.06 ± 0.38	20.86 ± 0.42
Never	23.95 ± 2.50	13.59 ± 2.01	33.35 ± 2.76	7.82 ± 1.57	21.30 ± 2.40
**Eggs**					
Frequently	15.34 ± 1.31	3.64 ± 0.68	24.00 ± 1.55	43.64 ± 1.80	13.37 ± 1.24
Occasionally	25.25 ± 0.50	11.75 ± 0.37	21.42 ± 0.47	20.77 ± 0.46	20.82 ± 0.46
Never	0.13 ± 0.07	51.19 ± 0.96	30.39 ± 0.88	0.00 ± 0.00	18.28 ± 0.74
**Fish**					
Frequently	19.61 ± 1.73	5.24 ± 0.97	32.05 ± 2.03	26.12 ± 1.91	16.98 ± 1.64
Occasionally	25.79 ± 0.53	10.25 ± 0.37	22.87 ± 0.51	19.69 ± 0.48	21.40 ± 0.50
Never	5.03 ± 0.36	42.07 ± 0.80	24.24 ± 0.70	11.69 ± 0.52	16.98 ± 0.61
**Chicken**					
Frequently	3.94 ± 1.25	14.37 ± 2.26	21.35 ± 2.64	34.29 ± 3.06	26.05 ± 2.83
Occasionally	24.73 ± 0.49	11.20 ± 0.36	23.44 ± 0.48	20.75 ± 0.46	19.88 ± 0.45
Never	4.19 ± 0.36	44.85 ± 0.89	24.76 ± 0.80	7.45 ± 0.47	18.76 ± 0.70
**Nuts**					
Frequently	5.15 ± 0.77	31.68 ± 1.62	13.76 ± 1.20	31.33 ± 1.61	18.09 ± 1.34
Occasionally	20.25 ± 0.44	18.91 ± 0.43	23.51 ± 0.46	17.02 ± 0.41	20.32 ± 0.44
Never	16.56 ± 0.85	24.16 ± 0.98	29.25 ± 1.04	12.39 ± 0.76	17.65 ± 0.87
**Fats**					
Never	28.86 ± 1.47	0.00 ± 0.00	59.02 ± 1.60	12.11 ± 1.06	0.00 ± 0.00
Occasionally	35.93 ± 0.72	2.43 ± 0.23	46.74 ± 0.75	13.91 ± 0.52	0.99 ± 0.15
Frequently	3.60 ± 0.24	38.01 ± 0.64	0.61 ± 0.10	20.70 ± 0.53	37.08 ± 0.63
**Sugar**					
Never	48.32 ± 3.61	1.83 ± 0.97	29.29 ± 3.29	0.31 ± 0.40	20.24 ± 2.90
Occasionally	45.48 ± 0.88	3.77 ± 0.34	25.09 ± 0.77	6.69 ± 0.44	18.97 ± 0.69
Frequently	6.71 ± 0.28	28.17 ± 0.51	23.08 ± 0.48	22.05 ± 0.47	19.98 ± 0.45
**Fried foods**					
Never	22.03 ± 2.11	14.56 ± 1.80	42.05 ± 2.51	6.65 ± 1.27	14.71 ± 1.80
Occasionally	19.48 ± 0.40	20.69 ± 0.41	22.68 ± 0.42	17.07 ± 0.38	20.08 ± 0.41
Frequently	7.96 ± 0.84	23.61 ± 1.32	27.16 ± 1.38	23.30 ± 1.31	17.97 ± 1.19
**Junk foods**					
Never	19.11 ± 0.72	19.96 ± 0.73	27.85 ± 0.81	14.54 ± 0.64	18.55 ± 0.71
Occasionally	18.73 ± 0.44	21.04 ± 0.46	22.28 ± 0.47	17.72 ± 0.43	20.23 ± 0.45
Frequently	4.03 ± 1.23	21.01 ± 2.55	21.17 ± 2.56	36.78 ± 3.02	17.01 ± 2.35
**Sweets**					
Never	19.84 ± 1.45	22.36 ± 1.51	31.43 ± 1.68	7.74 ± 0.97	18.64 ± 1.41
Occasionally	18.77 ± 0.39	20.75 ± 0.41	22.77 ± 0.42	17.92 ± 0.38	19.80 ± 0.40
Frequently	9.66 ± 1.43	17.88 ± 1.86	33.41 ± 2.29	19.74 ± 1.93	19.31 ± 1.92
**Aerated**					
Never	23.82 ± 1.12	20.12 ± 1.05	26.45 ± 1.16	10.76 ± 0.81	18.85 ± 1.03
Occasionally	18.04 ± 0.40	20.80 ± 0.42	23.21 ± 0.44	17.95 ± 0.40	20.00 ± 0.41
Frequently	7.98 ± 1.51	22.12 ± 2.31	27.85 ± 2.49	27.40 ± 2.48	14.65 ± 1.97
**Total**	**18.50 ± 0.37**	**20.75** ± **0.38**	**23.77** ± **0.40**	**17.29** ± **0.36**	**19.70** ± **0.38**

**Table 3.1:** Distribution of metabolic syndrome within five identified clusters among adolescents aged 10–19 years in India (2016-18)

	Prevalence (Std. Err.)			
	Overweight/Obesity	Lipid Anomaly	Pre-diabetes/Diabetes	Hypertension
**Cluster 1**	9.49 (0.65)	68.09 (1.03)	13.00 (0.74)	2.07 (0.31)
**Cluster 2**	13.74 (0.72)	65.76 (0.99)	12.03 (0.68)	2.66 (0.33)
**Cluster 3**	15.74 (0.71)	70.14 (0.89)	10.01 (0.58)	3.07 (0.34)
**Cluster 4**	10.93 (0.71)	71.52 (1.03)	9.81 (0.68)	2.45 (0.35)
**Cluster 5**	10.97 (0.67)	71.17 (0.97)	10.38 (0.65)	4.08 (0.42)
**Chi-square test**	32.04***	21.14***	47.72***	2.00
**Total**	**12.40 (0.31)**	**69.29 (0.44)**	**11.02 (0.30)**	**2.89 (0.16)**

***p-value < 0.01; **p-value < 0.05; *p-value < 0.10

**Table 3.2:** Significance (p-value) of chi-square statistics of association between metabolic syndrome within each identified cluster among adolescents aged 10–19 years in India (2016-18)

	Pearson Chi-square values (p-values)			
	Overweight/Obesity	Lipid Anomaly	Pre-diabetes/Diabetes	Hypertension
**Cluster 1**	6.28 (0.012)	9.31 (0.002)	29.83 (< 0.001)	0.55 (0.459)
**Cluster 2**	1.70 (0.192)	2.94 (0.086)	17.22 (< 0.001)	1.03 (0.311)
**Cluster 3**	23.67 (< 0.001)	0.44 (0.510)	1.51 (0.220)	0.59 (0.441)
**Cluster 4**	0.84 (0.359)	8.95 (0.003)	5.83 (0.016)	0.01 (0.927)
**Cluster 5**	8.12 (0.004)	4.53 (0.033)	4.89 (0.027)	0.33 (0.564)
**Chi-square test**	32.04***	21.14***	47.72***	2.00

**Table 4.1:** Distribution of micronutrient deficiencies within five identified clusters among adolescents aged 10–19 years in India (2016-18)

	Prevalence (Std. Err.)					
	Vitamin D Deficits	Vitamin B12 Deficits	Vitamin A Deficits	Folate Deficits	Iron Deficits	Zinc Deficits
**Cluster 1**	22.09 (0.91)	19.95 (0.88)	13.10 (0.74)	21.14 (0.90)	12.79 (0.74)	23.12 (0.93)
**Cluster 2**	22.28 (0.87)	27.47 (0.93)	6.96 (0.53)	34.64 (0.99)	17.72 (0.79)	26.18 (0.91)
**Cluster 3**	17.71 (0.74)	21.44 (0.80)	8.86 (0.55)	33.15 (0.91)	17.65 (0.74)	28.11 (0.87)
**Cluster 4**	23.01 (0.96)	26.35 (1.00)	9.73 (0.68)	30.40 (1.05)	17.69 (0.87)	24.76 (0.98)
**Cluster 5**	24.98 (0.92)	26.93 (0.95)	11.46 (0.68)	33.41 (1.01)	14.27 (0.75)	28.29 (0.96)
**Chi-square test**	25.03***	57.25***	58.16***	101.00***	42.57***	15.32**
**Total**	**21.82 (0.39)**	**24.34 (0.41)**	**09.91 (0.28)**	**30.81 (0.44)**	**16.12 (0.35)**	**26.24 (0.42)**

***p-value < 0.01; **p-value < 0.05; *p-value < 0.10

**Table 4.2:** Significance (p-value) of chi-square statistics of association between micronutrient deficiencies within each identified cluster among adolescents aged 10-19 years in India (2016-18)

	Pearson Chi-square values (p-values)					
	Vitamin D Deficits	Vitamin B12 Deficits	Vitamin A Deficits	Folate Deficits	Iron Deficits	Zinc Deficits
**Cluster 1**	23.22 (< 0.001)	34.09 (< 0.001)	0.14 (0.706)	23.66 (< 0.001)	15.22 (< 0.001)	5.84 (0.016)
**Cluster 2**	30.99 (< 0.001)	31.84 (< 0.001)	7.66 (0.006)	0.24 (0.625)	24.96 (< 0.001)	2.27 (0.132)
**Cluster 3**	8.01 (0.005)	2.79 (< 0.095)	1.94 (0.164)	6.99 (0.008)	0.95 (0.330)	8.08 (0.003)
**Cluster 4**	9.43 (0.002)	2.22 (0.136)	0.11 (0.743)	41.04 (< 0.001)	6.15 (0.013)	1.88 (0.170)
**Cluster 5**	0.59 (0.444)	0.18 (0.673)	22.01 (< 0.001)	54.33 (< 0.001)	5.68 (0.017)	0.23 (0.632)
**Chi-square test**	25.03***	57.25***	58.16***	101.00***	42.57***	15.32**

Based on the results, phenotypic traits of each individual’s diet behaviour within a cluster, cluster labels along with p-values of chi-squared statistics for association between being in a particular cluster and suffering from metabolic disorders or micronutrient deficiencies were provided as follows:

Cluster 1, referred to as “comparatively healthy” as individuals here were found consuming all seventeen items in moderation paired with prevalence of frequently consuming sugar was only 9.66% (SD = 1.43) which was relatively low among them compared to other clusters. This very cluster constitutes 19% of the total sample. Individuals in this clusters were found to have diabetes (p = < 0.001, chi-square statistics for association between being in cluster 1 and had diabetes was statistically significant at 0.1% level of significance) with the maximum prevalence of 13.00 (SD = 0.74) in comparison to other clusters. Further majorities were vitamin A (p = 0.706, not significant) and D deficit (p < 0.001, significant at 0.1% level of significance) with the prevalence of 13.10 (SD = 0.74) and 22.09 (SD = 0.91) respectively.

Cluster 2, another group perhaps classified as “Plant-based”, comprised of 21% of the clustered individuals. These individuals were characterised by less non-vegetarian diet consumption and mainly rely on vegetarian diet paired with frequent fats (38.01; SD = 0.64) and sugar (28.17; SD = 0.51) consumption. These individuals were mainly deficits of vitamin B12 (27.47, SD = 0.93; p < 0.001, significant at 0.1% significance level), D (22.28; SD = 0.87; p < 0.001, significant at 0.1% level of significance), folate (34.64; SD = 0.99; p = 0.625, chi-square statistics not significant), and iron (17.72; SD = 0.79; p < 0.001, significant at 0.1% significance level). Further two components of metabolic syndrome i.e., overweight/obesity (13.74; SD = 0.72; p = 0.192, not significant) and pre-diabetes/diabetes (12.03; SD = 0.68; p < 0.001, significant at 0.1% level of significance)) were quite prevalent among them.

Cluster 3, referred to as “Obesogenic diet”, individuals were prudent in terms of consuming high caloric diet, comprised of 24% of the total sampled adolescents. Here, majority individuals were seen consuming less cereals (40.89; SD = 5.98), pulses (42.35; SD = 4.22), greens (39.64; SD = 3.79), vegetables (39.86; SD = 3.51), fruits (33.35; SD = 2.76) paired with frequent fried foods (27.16; SD = 1.38), sweets (33.41; SD = 2.29), and aerated drinks (27.85; SD = 2.49). Majorities tend to have deficiencies like folate (33.15; SD = 0.91; p = 0.008, significant at 1% level of significance), iron (17.69; SD = 0.74; p = 0.330, not significant) and suffer from overweight/obesity (15.74; SD = 0.71; p < 0.001, significant at 0.1% level of significance), lipid anomalies (70.14; SD = 0.89; p = 0.510, not significant), and pre-hypertension/hypertension (3.07; SD = 0.34; p = 0.441, not significant). However, the association between cluster 3 and status of pre-diabetes/diabetes was statistically significant at 0.1% level of significance (p < 0.001) despite the fact that the prevalence of pre-diabetes/diabetes was less.

Cluster 4, another cluster which can be referred to as “Western”. This very cluster comprise of roughly 17% of the total sampled population. These individuals were found to have frequent junk (36.78; SD = 3.02) and aerated drinks (27.40; SD = 2.48) paired with frequent non-vegetarian diet (eggs: 43.64; SD = 1.80; fish: 26.12; SD = 1.91; and chicken: 34.29; SD = 3.06) consumption. These individuals were found to have lipid anomalies (71.52; SD = 1.03; p = 0.003, significant at 5% level of significance) paired with vitamin B12 (26.35; SD = 1.00; p = 0.136, not significant), D (23.01; SD = 0.96; p = 0.002, significant at 1% level of significance), and iron (17.69; SD = 0.87; p = 0.013, significant at 5% level of significant) deficiencies. However, the association between cluster 4 and status of pre-diabetes/diabetes (p = 0.003) was statistically significant at 1% level of significance.

Cluster 5, adolescents in this cluster were referred to as “convenient” and constitutes roughly 20% of the study sample. This cluster tend to eat all food groups occasionally. And further tend to be suffering from three components of metabolic syndrome i.e., lipid anomalies (71.17; SD = 0.97; p = 0.033, significant at 5% level of significance), pre-diabetes/diabetes (10.38; SD = 0.65; p = 0.027, significant at 5% level of significance), and pre-hypertension/hypertension (4.08; SD = 0.42; p = 0.564, not significant) along with vitamin A (11.46; SD = 0.68; p < 0.001, significant at 0.1% level of significance), B12 (26.93; SD = 0.95; p < 0.001, significant at0.1% level of significance), D (24.98; SD = 0.92; p = 0.444, not significant), folate (33.41; SD = 1.01; p < 0.001, significant at 0.1% level of significance), iron (14.27; SD = 0.75; p = 0.017, significant at 5% level of significance), and zinc (28.29; SD = 0.96; p = 0.632, not significant) deficiencies. However, the association between the cluster and the status of overweight/obesity was significant at 1% level of significance (p = 0.004) despite the fact that prevalence of it was low among those with convenient phenotype.

## Discussion

Since the number of people suffering from diet-related NCDs has been steadily rising. Proportion of individuals consuming energy dense food and foods lacking nutrients of concern (termed as obesogenic diet such as packaged foods which are high in unhealthy fat, salt, and sugar) seen particularly among adolescents is on rise. Hence, making them prone to have early onset of metabolic syndrome and CVDs. Further, the present study elaborated on how equating various food groups consumed by individuals regularly with their health status is important. Which is sufficient to anticipate the risk of metabolic syndrome, even among younger ages. However, the existing clinical practices which need to be modified, lacked this particular aspect where individuals were told what not to eat, but what was required was to know what dietary practices they follow on a regular basis, hence, making informed decisions. Therefore, the present study was needed to identify clusters and ascertain the consumption pattern of certain obesogenic dietary habits (or food groups) which were association with metabolic syndrome and micronutrient deficiencies among adolescents.

Present study identified five disjoint clusters based on consumption of seventeen food items using k-means clustering algorithm. Further, phenotypic characteristics were analysed based on individuals’ consumption pattern of certain food groups. Clusters were named based on how prudent are these in terms of consuming an unhealthy diet. However, similar approach can be seen adopted by previously done studies but either in countries other than India or at subnational level [29, 30]. Nevertheless, the present study is the first in India among adolescents with cluster phenotype. It has been found that, individuals following a highly prudent obesogenic diet had a significant association with two major components of metabolic syndrome i.e., obesity and diabetes. Results obtained from the present study were seen aligned with results obtained in other studies [28]. However, it has been found that those who were in highly obesogenic cluster, with less cereal, milk and fruits consumption, were more prone to develop obesity, lipid anomalies, pre-diabetes/diabetes and pre-hypertension/hypertension, similar findings has been reported by other studies [2, 9, 31]. Which was evident from the clinical perspective that there exist a profound relationship and pathways between obesity, MetS, and CVDs [32]. In a recent study, it has been found that the prevalence of MetS among adolescents in India was clustered among those living in urban place of residence [33]. Perhaps due to the availability, accessibility, and, affordability of high caloric, low nutrient-dense foods paired with physical-inactivity [10, 34, 35].

The present study further highlights that cluster of individuals classified as western diet were majorly suffering from lipid anomalies. That is, individuals who were consuming animal-based diet i.e., high-protein but less fibre, they were majorly suffering from micronutrient deficiencies and lipid anomalies. Which can be explained as they were relying more on animal-based protein. Whereas a human body requires greens in their diets, a lack of which causes impaired glucose tolerance and an abnormal lipid profile [36–38]. Similar relationship between being non-vegetarian and suffering from high cholesterol were seen in other studies [36, 39]. Further those who were called as ‘convenient’ were found to consume all food groups occasionally which means at the end of the day, they were consuming more calories than required. As, convenient pattern can be associated with high intake of carbohydrate, sugar, and fats. Hence found suffering from three components of metabolic syndrome i.e., lipid anomalies, pre-diabetes/diabetes, and pre-hypertension/hypertension. Similar cluster was found reported in studies done previously [8, 29, 40]. It can also be seen that the largest cluster that was formed were of those who consumed obesogenic diet. Which means, roughly every fourth adolescent aged individual followed an obesogenic diet.

Another interesting finding from the present study would be that despite there were few clusters which were showing high prevalence of pre-hypertension/hypertension but the association between each cluster with the status of the condition was found statistically insignificant. However, it has been found that there was a profound that parental family history of hypertension played an important role in predicting hypertension among adolescents [41]. According to the study’s findings, clinical practitioners can anticipate the high risk of any of the four components of metabolic syndrome by merely reading individuals’ dietary practices except for the status of hypertension. However, individuals physical in/activity and parental family history of MetS would be a holistic approach, which was missing in the present study due to insufficient information on the same. Therefore, enabling informed clinical decisions to improve dietary practices that an individual was following might result in reverting and perhaps avoiding the early onset of metabolic syndrome among them.

As in almost all the clusters there was a significant consumption of fried, junk, aerated drinks high in sugar contents, it can be seen as an opportunity to actually introduce food labels, particularly front-of-package-labels (FOPL) with a “warning” if any packaged item is high in saturated fats, sugar, and oils [2, 42]. Hence ensuring people are aware of what they are consuming. Availability of such food groups cannot be controlled but consumer behaviour towards availing such foods can be restricted by making them aware how such food groups affects human body in terms of health [8]. Besides, in a study, it has been found that FOPL do influence the intention to purchase packaged food, and ‘warning’ labels were found to be the most effective FOPL in helping Indian consumers to identify unhealthy food [42]. However, apart from consumers, Public Policymakers are the ones who should facilitate consumers with consumables such as packaged foods high in saturated fats, sugar, and salt released under proper guidelines and closely monitor the manufacturers and suppliers of such consumables. As it is a pertinent issue in India right now. Lastly, technological enhancement towards generating new tools for detecting diseases in the food, which would make the process faster is required. Which involves the clever use genetic tricks to find the disease-causing germs in the food hence a smart and efficient way of diagnosing and preventing foodborne illnesses [43].

### Strengths and limitations

Since the study is based on a nationally representative survey data among children and adolescents aged 10–19 years hence, results obtained can be generalized at a national level. Furthermore, this study is the first in India in ascertaining clusters of individuals in accordance with what all food groups they were consuming and how it is associated with metabolic syndrome and other micronutrient deficiencies. Limitations comes with a fact that the study is based on secondary dataset hence cannot establish the causation between obtained clusters and metabolic syndrome or micronutrient deficiencies. Further, the items used for cluster analysis were self-reported which could be different from the actual scenario. Hence, there could be a potential recall bias in reporting how frequently an individual is consuming a certain food item. Lastly, study is based on a cross sectional data hence, unable to determine for how long they have been following a certain eating habit which might help in determining the extent of exposure that would result in such health outcomes.

#### Future directions

It was evident from the limitations of the study that for future research, there is still a room for exploring how is the causal relationship between the obesogenic diet and metabolic syndrome among adolescents in India. Is it similar to that of adults or vice-versa? Further, how individuals in different phenotypic clusters would behave if given intervention that would improve the health status of these individuals? How is physical inactivity and parental family history of MetS play an important role in predicting the same among adolescents?

## Conclusions

The present study identified five heterogeneous clusters which provides valuable insight into the dietary habits of the study sample and can inform public health interventions aimed at promoting healthy eating behaviours and reducing the risk of MetS. It has been found that every fourth sample unit was following a highly prudent obesogenic diet which calls for an immediate action. Adolescents in India show a strong association between obesogenic diet and metabolic syndrome. Therefore, the burden of metabolic syndrome at early ages can be prevented by controlling obesogenic dietary practices and addressing micronutrient deficiencies. This may be done by targeted health promotional campaigns in schools and college-going populations in India. Hence, in a way controlling early onset of complicated cardiometabolic illnesses.

Further, consumer behaviour towards availing packaged and junk foods which are high in saturated fat, oil, sugar, and salt can be restricted by introducing warning label on packaged and processed foods. Educating adolescents on the importance of healthy eating habits is crucial in preventing and managing obesity and metabolic syndrome. Therefore, public health interventions can be tailored to meet the unique needs of different populations and improve overall health outcomes by targeting specific dietary patterns.

Tables.

## Data Availability

The CNNS data can be accessed from the Population Council (Delhi, India) upon request. The report and survey tools are available in the public domain on the following website: https://nhm.gov.in/New_Updates_2018/resources/CNNS_reports.zip and data can be obtained on request from cnns.pc@gmail.com.

## List of abbreviations

MetS: Metabolic Syndrome
CVDs: Cardiovascular diseases
NCDs: Non communicable diseases
CNNS: Comprehensive National Nutrition Survey
BMI: Body mass index
HbAc1: Glycated Haemoglobin
LDL: Low density lipoprotein
HDL: High density lipoprotein
FOPL: Front-of-packet labels
